# Impact of acute ethanol intake on cardiac autonomic regulation

**DOI:** 10.1038/s41598-021-92767-y

**Published:** 2021-06-24

**Authors:** Stefan Brunner, Raphaela Winter, Christina Werzer, Lukas von Stülpnagel, Ina Clasen, Annika Hameder, Andreas Stöver, Matthias Graw, Axel Bauer, Moritz F. Sinner

**Affiliations:** 1 Department of Medicine I, University Hospital, Ludwig Maximilians University (LMU) Munich, Ziemssenstrasse 1, 80336 Munich, Germany; 2 German Center for Cardiovascular Research (DZHK), Partner Site: Munich Heart Alliance, Munich, Germany; 3 Institute of Legal Medicine, LMU Munich, Munich, Germany; 4 Department of Internal Medicine III, Medical University Innsbruck, Innsbruck, Austria

**Keywords:** Cardiology, Medical research

## Abstract

Acute alcohol consumption may facilitate cardiac arrhythmias underlying the ‘Holiday Heart Syndrome’. Autonomic imbalance is promoting atrial arrhythmias. We analyzed the effects of alcohol on measures of the cardiac autonomic nervous system and their relation to arrhythmias. In 15 healthy individuals, alcohol was administered parenterally until a breath alcohol concentration of 0.50 mg/l. High-resolution digital 30-min ECGs were recorded at baseline, at the time of maximum alcohol concentration, and after alcohol concentration returned to near baseline. Using customized software, we assessed periodic repolarization dynamics (PRD), deceleration capacity (DC), standard measures of heart rate variability (SDNN; RMSSD; LF; HF), and standard ECG parameters (mean heart rate; PQ; QRS; QTc interval). At the maximum alcohol concentration, PRD levels were significantly increased compared to baseline [1.92 (IQR 1.14–3.33) deg^2^ vs. 0.85 (0.69–1.48) deg^2^; p = 0.001]. PRD levels remained slightly increased when alcohol concentrations returned to baseline. DC levels were significantly decreased at the maximum alcohol concentration compared to baseline [7.79 (5.89–9.62) ms vs. 9.97 (8.20–10.99) ms; p = 0.030], and returned to baseline levels upon reaching baseline levels of alcohol. Standard HRV measures were reduced at maximum alcohol concentration. The mean heart rate increased significantly during alcohol administration. QRS and QTc duration were significantly prolonged, whereas PQ interval showed no change. Our findings revealed an increase of sympathetic activity and a reduction of parasympathetic activity under the influence of alcohol administration, resulting in autonomic imbalance. This imbalance might ultimately trigger arrhythmias underlying the ‘Holiday Heart Syndrome’.

## Introduction

Acute excessive alcohol consumption (“binge drinking”) may cause cardiac rhythm disturbances in otherwise healthy individuals, often referred to as ‘Holiday Heart Syndrome’. Both ventricular and supraventricular arrhythmias, predominantly atrial fibrillation, have been described^1^. The underlying mechanisms of arrhythmogenesis are incompletely understood.

An alcohol-induced imbalance of the autonomic nerve system (ANS) may be a potential contributor to arrhythmogenesis. In the Munich BREW study, we recently demonstrated in over 3,000 visitors of the Munich Octoberfest that acute alcohol consumption results in both an increase of sinus tachycardia and a reduction of respiratory sinus arrhythmia^2,3^. Both findings suggest an autonomic imbalance. Measures of heart rate variability (HRV) assessing the physiological beat-to-beat variation of the heart are intended to quantify the ANS. However, standard HRV measures are more or less influenced by both, the sympathetic and parasympathetic branches of the ANS. Several prior studies investigated the effects of different levels of acute and chronic alcohol intake on HRV measures. These studies indicated an influence of alcohol consumption, particularly on HRV measures indicating reduced vagal nerve modulation^4^. Most recently, a prospective study in binge drinkers equipped with holter ECG monitors found very similar findings^5^.

More recently, novel electrocardiogram (ECG)-based measures have been developed to specifically quantify the activation levels of the parasympathetic and sympathetic branches of the ANS, respectively. Deceleration capacity (DC) is an advanced and robustly validated marker of HRV, predominantly reflecting parasympathetic activity of the ANS. DC is an integral measure of all deceleration-related oscillations of heart rate, including regulations in the very low, low, and high frequency bands^6^. In clinical trials, an impaired DC predicted late mortality after myocardial infarction. The predictive performance exceeded that of abnormal standard HRV measures^6,7^. In contrast to measures of HRV, the ECG-based marker Periodic Repolarization Dynamics (PRD) quantifies dynamic properties of the T-wave. More precisely, PRD captures low frequency oscillations T-wave vector changes, which are believed to be caused by phasic activation of the efferent sympathetic nervous system. Recent studies in heart failure patients could demonstrate that increased PRD was strongly associated with ventricular arrhythmias and sudden death^8–10^.

In the present study, we therefore aimed to investigate variation of advanced ANS markers DC and PRD in the stetting of standardized acute alcohol exposure.

## Methods

### Study population

We enrolled 15 healthy volunteers (8 male, 7 female) with a mean age of 28.8 ± 6.8 years (range 19–45 years). Both known heavy drinkers and total alcohol abstainers were excluded. We further excluded individuals with a history of cardiovascular, cerebrovascular, respiratory, and infectious diseases, those with an implanted pacemaker or defibrillator, and those using any medication. All individuals provided written informed consent. The study protocol was approved by the Ethics Committee of the Ludwig-Maximilians-University of Munich, and conforms to the principles outlined in the Declaration of Helsinki. All data underlying this article are available in this article.

### Alcohol administration

**Figure 1:**
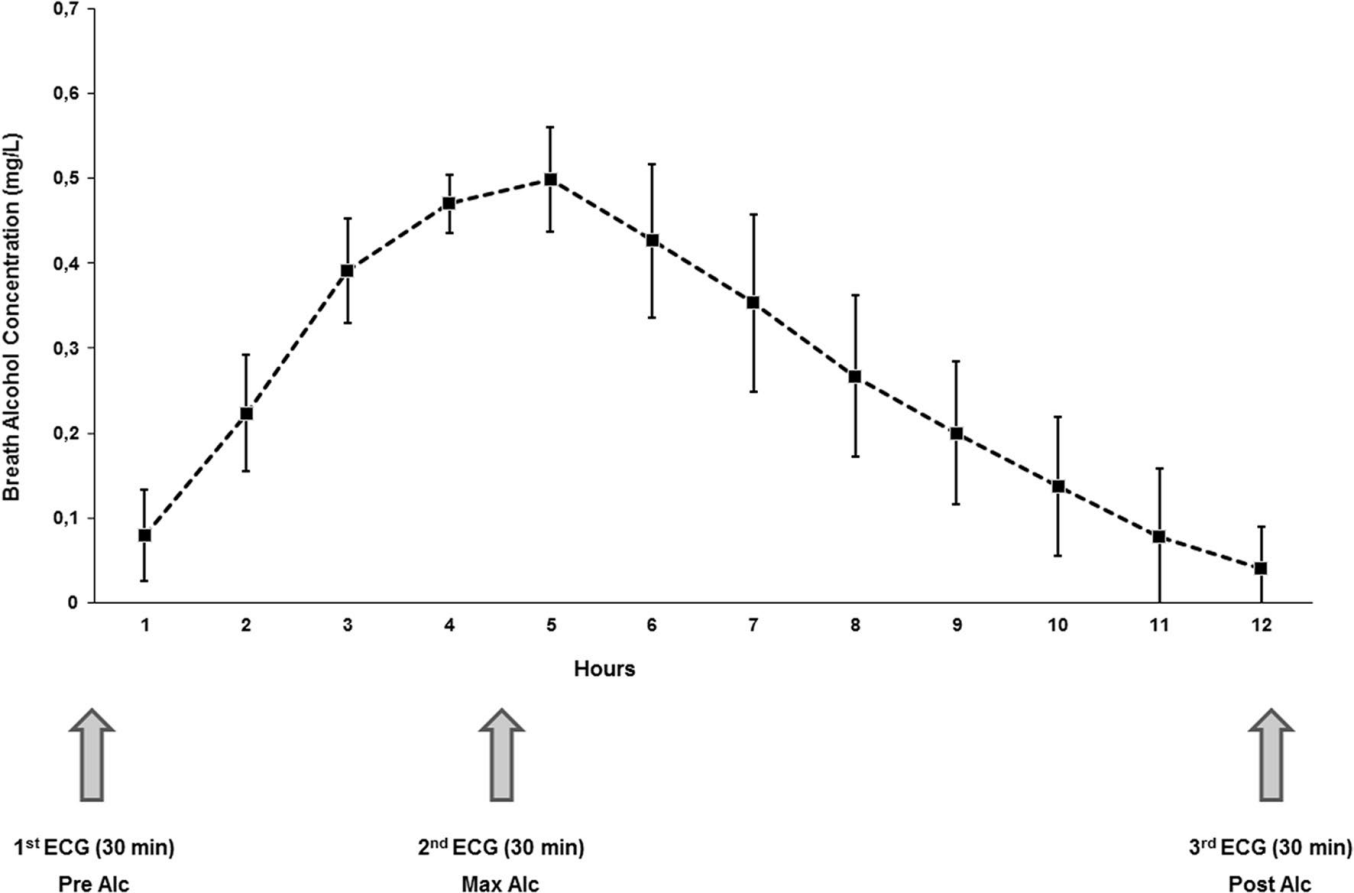
Study design and mean breath alcohol concentration during the trial. 30-min-ECGs were recorded before starting the alcohol infusion, at the time of maximum breath alcohol concentration, and after alcohol concentration dropped to approximately 0.05 mg/l. N = 15 for all experiments and analyses.

To ensure standardized study conditions, alcohol was administered intravenously using a 7% ethanol/ 5% glucose solution. The flow rate of the infusion was controlled via an infusion pump and was set at 0.40 g alcohol / kg body weight / hour in males and 0.33 g alcohol / kg body weight / hour in females. The target breath alcohol concentration of 0.50 mg/l was reached after 3.5–4 h. Subsequently, the flow rate of the infusion was reduced to 0.105 g alcohol / kg body weight / hour in males and 0.090 g alcohol / kg body weight / hour in females in order to maintain the target breath alcohol concentration for the duration of 1 h (Fig. 1).

### Assessment of alcohol concentration

Breath alcohol concentration in milligram per liter (mg/l) was assessed every 15 min using a Dräger Alcotest 9510 handheld device (Drägerwerk AG, Lübeck, Germany). After stopping the alcohol infusion, measurements were continued until breath alcohol levels dropped to approximately 0.05 mg/l (Fig. 1). After reaching the target breath alcohol concentration of 0.50 mg/l, the measurement was independently verified by the assessment of blood alcohol values.

### Assessment of ECG-based measures of the cardiac ANS

In all individuals, a high-resolution digital 30-min-ECG (1000 Hz, Schiller, Baar, Switzerland) was performed in Frank leads configuration in supine and resting position under standardized conditions. Recordings were performed before starting the alcohol infusion as baseline measurement (Pre Alc), at the time of maximum alcohol concentration (Max Alc), and after breath alcohol concentrations dropped to approximately 0.05 mg/l (Post Alc) (Fig. 1). ECG raw signals were pre-processed by an experienced technician. Heart rate, PRD, DC, and standard measures of HRV were assessed as previously published using customized software^6,9^.

Briefly, for the calculation of PRD, the spatio-temporal characteristics of each T wave were mathematically integrated into a single vector T°. The angel dT° between two successive repolarization vectors was calculated and displayed over time, representing the spontaneous degree of instantaneous repolarization instability. Typically, a low-frequency periodic augmentation of dT° can be observed. The spectral properties of the dT° signal were quantified by means of continuous wavelet transformation that provides wavelet coefficients for each scale at each time point. PRD was defined as the average wavelet coefficient corresponding to frequencies of 0.1 Hz or less^9^.

To determine DC, in a first step, beat-to-beat intervals longer than the preceding interval were defined as decelerating anchors. In a second step, segments around anchors were averaged to obtain the phase-rectified signal averaging (PRSA) signal. The central part of the PRSA signal was quantified by wavelet-analysis to obtain the numerical measure of DC^6^.

In addition, the following standard measures of HRV were assessed: The standard deviation of all NN intervals (SDNN) as an estimate of overall HRV and the square root of the mean of the sum of the squares of differences between adjacent NN intervals (RMSSD) as an estimate of short-term components of HRV. As spectral components, we calculated low frequency (LF) and high frequency (HF) components and their ratio (LF/HF).

### Statistical analyses

PRD, DC, and HRV measures are expressed by their medians and 25th and 75th percentiles. Differences between these measures were tested by the Wilcoxon signed-rank test. Standard ECG measures are expressed as means ± standard deviations. Differences between these measures were tested by the paired t-test. A two-sided alpha error of p < 0.05 was considered statistically significant.

### Data availability

All data are incorporated into the article.

## Results

### Time course of breath alcohol concentration

The mean maximum breath alcohol concentration was 0.50 ± 0.05 mg/l. This maximum alcohol concentration was reached after 3.9 ± 0.7 h on average. The confirmatory blood alcohol concentration measured after reaching the target breath alcohol concentration was 1.06 ± 0.19 ‰. The mean study duration from starting the alcohol infusion until the breath alcohol concentration dropped to approximately 0.05 mg/l was 10.4 ± 1.3 h (Fig. 1).

### dT° signal and periodic repolarization dynamics (PRD)

**Figure 2:**
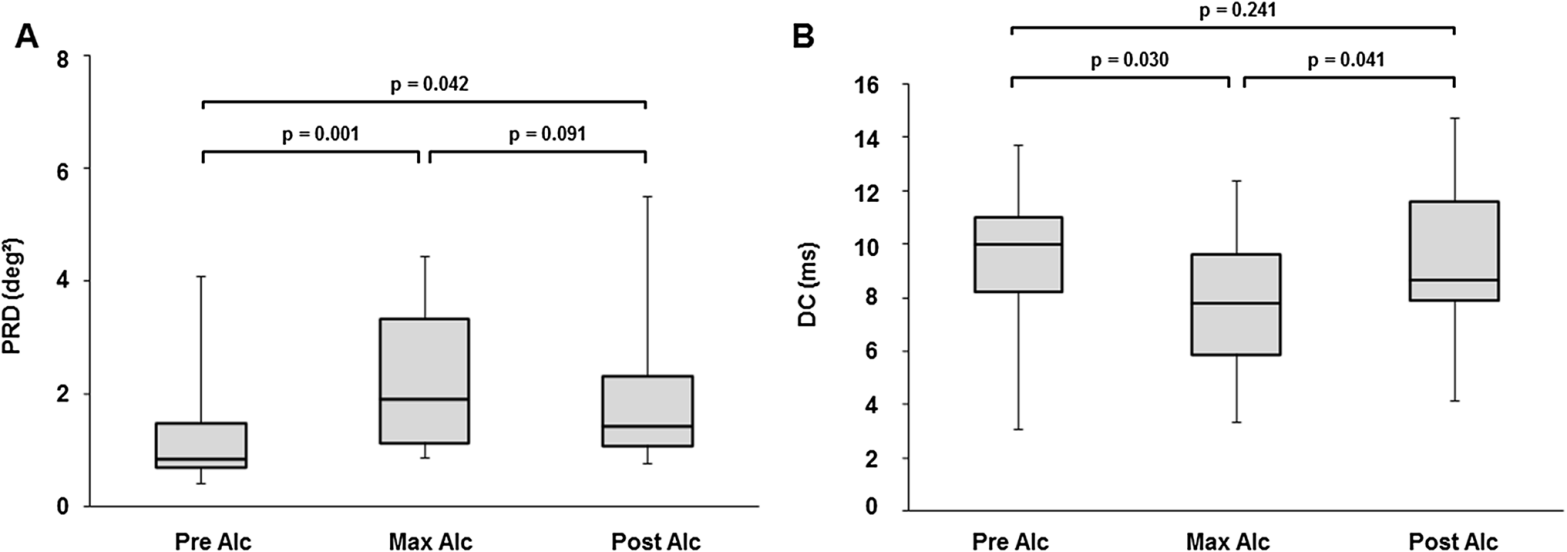
Median levels of (**A**) periodic repolarization dynamics (PRD) and (**B)** deceleration capacity (DC) before starting the alcohol infusion (Pre Alc), at the time of maximum breath alcohol concentration (Max Alc), and after alcohol concentration dropped to approximately 0.05 mg/l (Post Alc). Error bars indicate the interquartile range. One outlier at Max Alc (PRD = 10.19 deg^2^) not depicted. N = 15 for all experiments and analyses. Differences were tested by the Wilcoxon signed-rank test.

The median Pre Alc PRD level at baseline was 0.85 deg^2^ [interquartile range (IQR) 0.69 deg^2^; 1.48 deg^2^]. Compared to baseline, the median Max Alc PRD level at the time of maximum alcohol concentration significantly increased to 1.92 deg^2^ [IQR 1.14 deg^2^; 3.33 deg^2^], (p = 0.001). Median Post Alc PRD levels decreased to 1.44 [IQR 1.09 deg^2^; 2.31 deg^2^] (p = 0.09) after breath alcohol concentration dropped to 0.05 mg/l. Compared to Pre Alc levels, Post Alc measures remained significantly increased (p = 0.042) (Fig. 2A).

### Changes of the deceleration capacity (DC)

The median Pre Alc DC level at baseline was 9.97 ms [IQR: 8.20 ms; 10.99 ms]. Compared to baseline, the median Max Alc DC level at the time of maximum alcohol concentration was significantly decreased to 7.79 ms [IQR: 5.89 ms; 9.62 ms] ms, (p = 0.030). From the maximum alcohol concentration, the median Post Alc DC level significantly increased to 8.66 ms [IQR: 7.89 ms; 11.59 ms] (p = 0.041) after breath alcohol concentration dropped to 0.05 mg/l, approximating the baseline level (p = 0.24) (Fig. 2B).

### Standard measures of heart rate variability (HRV)

SDNN, RMSSD, LF, and HF decreased from baseline to the time of maximum alcohol concentration, and slightly increased after alcohol levels returned towards normal without reaching baseline levels. The LF/HF ratio increased from baseline to the time of maximum alcohol concentration and remained at that level thereafter. Table 1 summarizes all median values and statistics of standard HRV measures.

**Table 1:** Standard HRV parameters.

	Pre Alc	Max Alc	Post Alc	p-value Pre vs. max	p-value Max. vs. post	p-value Pre vs. post
SDNN, ms	62.4 (50.2–70.5)	51.8 (44.2–63.4)	59.9 (47.1–66.1)	0.326	1.000	0.135
RMSSD, ms	39.6 (33.0–50.4)	24.7 (17.3–37.6)	28.3 (22.2–31.5)	0.005	0.583	0.005
LF, ms^2^	771.7 (529.5–1239.6)	620.6 (276.2–987.6)	724.2 (479.4–1252.8)	0.030	0.119	0.358
HF, ms^2^	436.1 (192.1–965.8)	181.4 (52.5–423.3)	173.4 (125.8–288.7)	0.009	1.000	0.002
LF/HF ratio	1.71 (1.29–4.61)	3.26 (1.74–5.19)	3.22 (2.00–5.83)	0.326	0.279	0.002

N = 15 for all experiments and analyses. Differences were tested by the Wilcoxon signed-rank test.

*HRV* heart rate variability, *SDNN* standard deviation of NN intervals, *RMSSD* root mean square of successive differences, *LF* low frequency, *HF* high frequency, *Pre Alc* before alcohol intake, *Max Alc* maximum blood alcohol concentration, *Post Alc* after alcohol intake.

### Standard ECG parameters

Mean heart rate significantly increased from baseline to the time of maximum alcohol concentration and remained elevated thereafter. Likewise, mean QTc interval significantly increased from baseline to the time of maximum alcohol concentration and remained at that level. Mean QRS duration significantly increased from baseline to the time of maximum alcohol concentration, and significantly decreased thereafter. Mean PQ interval remained stable throughout study procedures (Table 2).

**Table 2:** Standard ECG parameters.

	Pre Alc	Max Alc	Post Alc	p-value Pre vs. max	p-value Max. vs. post	p-value Pre vs. post
MHR, min^−1^	66.5 ± 6.1	76.0 ± 9.4	75.2 ± 6.9	< 0.001	0.736	< 0.001
PQ, ms	157.1 ± 20.8	156.5 ± 22.7	155.9 ± 19.6	0.738	0.790	0.547
QRS, ms	97.7 ± 8.2	100.9 ± 8.7	98.1 ± 8.7	0.026	0.018	0.716
QTc, ms	392.2 ± 16.4	416.9 ± 16.5	412.0 ± 14.0	< 0.001	0.182	< 0.001

N = 15. Differences were tested by the paired t-test.

*Pre Alc* before alcohol intake, *Max Alc* maximum blood alcohol concentration, *Post Alc* after alcohol intake, *MHR* mean heart rate.

## Discussion

In our study, we investigated the influence of acute alcohol intake on novel ECG derived measures of the ANS. Maintaining highly standardized conditions, the observed changes of PRD and DC suggest significant effects of both the sympathetic and the parasympathetic branches of the ANS, indicating a significant ANS imbalance in response to acute alcohol exposure.

So far, the impact of acute alcohol intake on the ANS has primarily been assessed using standard HRV measures, most recently in a prospective cohort of binge drinkers equipped with holter ECG monitoring^5^. These measures reflect the overall sympathetic and parasympathetic activation of the ANS and are thus able to identify an ANS imbalance. Yet, none of these standard measures specifically quantifies either sympathetic or parasympathetic activation. In our study, we investigated novel ECG-derived measures of the ANS, which have been shown to specifically differentiate between sympathetic and parasympathetic activites^6,9^. Thus, these novel measures provide valuable insights into pathophysiologic changes to the ANS caused by acute alcohol exposure.

Further, prior studies applied varying settings of acute alcohol exposure^4^. Typically, alcohol was consumed orally in different quantities and various ways of preparation. In contrast, our study design ensured highly standardized conditions by administrating alcohol intravenously, targeting pre-specified breath and blood alcohol concentrations by individually tailoring the infusion rate. Hence, our study design minimizes potential bias caused by the type of consumed alcohol and by the mode of alcohol administration.

The first main finding of our study is a significant increase of PRD levels at the time of maximum alcohol concentration and a slight, but not significant decrease after alcohol concentration dropped to near baseline levels. This finding suggests an alcohol-induced activation of the sympathetic ANS branch. PRD levels ≥ 5.75 deg^2^ have been established as a strong predictor of late mortality in patients after a myocardial infarction^9^. Despite a significant increase in PRD levels following alcohol exposure in our study, only a single study participant reached a level exceeding 5.75 deg^2^, the threshold indicating an excess risk of mortality after myocardial infarction^9,11^. However, the clinical relevance of elevated PRD levels in healthy individuals has yet to be resolved.

The second main finding of our study is a significant decrease in DC levels, reflecting a reduced activity of the parasympathetic ANS branch. After alcohol concentrations dropped to near baseline levels, DC levels also returned to baseline values. Similar to PRD, DC was also established as a predictor of mortality in patients after myocardial infarction^6^. So far, several studies have investigated changes in DC levels under different physiologic and pathophysiologic conditions^12–14^. However, a clinical relevance of altered DC has only been substantiated in patients after myocardial infarction and in patients undergoing transcatheter aortic valve implantation^6,7,12^.

Taken together, our primary results clearly indicate that the alcohol induced imbalance of the ANS is caused by both an increase in sympathetic activity and a decrease in vagal tone. This is in line with a study analyzing skeletal muscle sympathetic activity after alcohol intake^15^. Studies on parasympathetic activity beyond standard HRV-based analyses are lacking.

Regarding standard HRV parameters, both time domain measures and frequency domain measures were reduced after acute alcohol intake^16^. This is in line with prior results investigating resting HRV following acute oral alcohol consumption. In these prior studies, all HRV measures were suppressed after acute alcohol consumption except for the low-frequency component, which was not consistent across studies^4^. These prior results indicated a general ANS imbalance following acute alcohol exposure, yet were unable to adjudicate the predominance of a specific ANS branch. Our results overcome this limitation by clearly demonstrating a specific sympathetic activation and parasympathetic suppression.

Further, we analyzed standard ECG parameters in relation to acute alcohol exposure. Mean heart rate was significantly increased at the time of maximum alcohol concentration. Prior studies also reported an increase in heart rate following alcohol intake in different settings as a response to an ANS imbalance^2,15,17,18^. Our current results now adjudicate this heart rate increase to be an effect of both sympathetic activation and parasympathetic withdrawal. We also observed a significant prolongation of QRS duration and of QTc intervals, findings in line with prior studies^19,20^. Our findings on PQ interval duration are less consistent. Whereas we did not observe a change in relation to alcohol exposure, a study by Lorsheyd et al. described a PQ prolongation after binge drinking^20^. Despite our highly standardized experimental setting, our cohort may have been too small to reflect minor changes.

The underlying pathophysiology linking acute alcohol exposure and our observed ECG changes remain incompletely understood. While the autonomic effects are commonly attributed to the effects of alcohol metabolites and acetaldehyde in particular^17,21^, less literature is available for the effect of alcohol on standard ECG measures like QRS duration and the QTc interval. In our cohort, the alcohol dependent prolongation of QRS duration was only marginal. We speculated that this subtle change may reflect a rate dependent prolongation rather than an influence of sympathetic activation. However, an increased QTc interval following alcohol exposure has repeatedly been shown in binge drinkers^19,20^. Whereas the specific pathophysiologic link has yet to be demonstrated, it has been shown repeatedly that sympathetic activation results in QTc prolongation particularly when the sympathetic activation is not the result of physical exercise^22,23^. We hence speculate that the presumed underlying pathophysiology is similar for HRV measures and the QTc interval.

Putting our findings into context, we were able to detect distinct changes of advanced ECG-based measures showing both an alcohol-induced activation of the sympathetic branch and a reduction of the parasympathetic branch of the ANS. This autonomic imbalance may be the pathophysiologic background for the development of atrial and ventricular arrhythmias following excessive alcohol consumption^24^. This association of acute alcohol consumption with the occurrence of arrhythmias is well known as the ‘Holiday Heart Syndrome’ that was first described more than 40 years ago^1^.

Some limitations need to be considered when interpreting our study. Due to the highly experimental design, we were restricted to enrolling the presented number of participants only. We were thus limited by statistical power to identify modest effects and to identify relevant strata including sex-related differences. Our ECGs were analyzed at specific timepoints only. We thus cannot comment on the kinetics of alcohol-level-related changes. Finally, our results refer to acute alcohol exposure only. The relevance of chronic alcohol exposure on the ANS remains to be investigated elsewhere.

## Conclusions

We analyzed the effect of acute alcohol exposure on novel ECG-based HRV measures, which specifically quantify the sympathetic and parasympathetic activation of the ANS. Applying highly standardized study conditions, we clearly observe an increase of sympathetic activity and a reduction of parasympathetic activity in response to alcohol exposure. We speculate that this combination of autonomic imbalance lays the foundation for an acute alcohol-induced arrhythmogenic substrate that may lead to the ‘Holiday Heart Syndrome’.

## Data Availability

All data generated or analysed during this study are included in this published article.
